# Influence of the 6061 Aluminium Alloy Thermo-Viscoplastic Behaviour on the Load-Area Relation of a Contact

**DOI:** 10.3390/ma14061352

**Published:** 2021-03-11

**Authors:** André Rudnytskyj, Stefan Krenn, Georg Vorlaufer, Carsten Gachot

**Affiliations:** 1 AC2T research GmbH, Viktor-Kaplan-Straße 2/C, Wiener, 2700 Neustadt, Austria; stefan.krenn@ac2t.at (S.K.); georg.vorlaufer@ac2t.at (G.V.); 2 TU Wien, Institute of Engineering Design and Product Development, Lehárgasse 6, Objekt 7 (Hoftrakt BD, Campus Getreidemarkt), 1060 Wien, Austria; carsten.gachot@tuwien.ac.at

**Keywords:** contact model, viscoplasticity, temperature dependence, load–area relation, contact patch, material model, constitutive relations, finite element method, aluminium alloy, 6061

## Abstract

The contact between solids in metal-forming operations often involves temperature-dependent viscoplasticity of the workpiece. In order to estimate the real contact area in such contexts, both the topography and the deformation behaviour should be taken into account. In this work, a deterministic approach is used to represent asperities in appropriately shaped quadratic surfaces. Such geometries are implemented in indentation finite element simulations, in which the indented material has thermo-viscoplastic properties. By creating a database of simulation data, investigations in terms of contact load and area for the specifically shaped asperities allow for an analysis on the influence of the material properties on the load–area relation of the contact. The temperature and viscoplasticity greatly define how much load is supported by a substrate due to an indenting asperity, but the description of the deformation behaviour at small values of strain and strain rate is also relevant. The pile-up and sink-in regions are very dependent on the thermo-viscoplastic conditions and material model, which consequently affect the real contact area calculation. The interplay between carried load and contact area of a full surface analysis indicates the role that different sized asperities play in the contact under different thermomechanical conditions.

## 1. Introduction

Estimation of the real contact area between two contacting solids can be seen as an initial step towards investigating friction and wear of that contact, which are of uttermost interest in various engineering applications [1,2,3,4,5,6,7,8,9,10]. In the context of metalworking, bulk forming operations such as hot rolling of Al alloys are greatly dependent on the contact conditions: the real area of contact partly defines the friction forces that move the workpiece through the roll bite [11], being thus a fundamental aspect of the process. Accurate prediction and control of friction in these processes are highly desirable, since they ultimately contribute to an optimised process in terms of energy consumption.

Although attempts to measure the real contact area have been explored in the literature [12], it is generally impractical to directly observe the contact itself. Hence, researchers have often turned to *contact models* to obtain information on the degree of contact (ratio between real contact area and nominal or apparent contact area). Contact model, in the sense used in this work, refers to a methodology for quantifying the real contact area in a contact between solid bodies. An appropriate contact model must take into account not only the topography of the surfaces and loading conditions but also the nature of the deformation of the materials involved. In metal forming, one of the major features of deformation of workpieces is the *thermo-viscoplastic* behaviour of the material, i.e., the interdependence between stress, strain, plasticity, time, and temperature in the deformation process. Such interplay lies within the scope of *creep*, defined as an inelastic time-dependent deformation when a material is subjected to a (constant or variable) stress at sufficiently high temperatures [13]. thermo-viscoplasticity causes certain aluminium alloys such as 6061 [14] to have significant changes in its flow stress $\sigma_{f}$ as the strain ε, strain rate $\dot{\varepsilon }$, and temperature *T* vary [15]. In view of this, a proper contact model in the metal-forming context should account for such thermo-viscoplastic parameters.

In the literature, *hardness* has been the primary translator between material and contact when plasticity is involved. In an indentation, it is well known that, when the mean pressure reaches a value of approximately 2.6–3 times the yield stress ($\sigma_{y}$) of the softer material, the material around the region of contact has plastically deformed. Therefore, the mean contact pressure in a so-called fully plastic indentation, i.e., the *indentation hardness H*, is expressed in terms of the yield stress of the deforming material as $H=c\sigma_{y}$, where *c*, sometimes named the “constraint factor”, varies between 2.6 and 3 (for aluminium, the value 2.8 is reported by Tabor [16]). The relation between hardness and contact area goes back to Bowden and Tabor [17,18], who assumed that the real contact area between two surfaces occurs at the tip of surface peaks, i.e., the tip of *asperities*. At these tips, the contact pressure would be high enough so that plastic flow would occur at the softer material, resembling plastic indentation as studied by Brinell and thoroughly discussed in the book of Tabor [16]. In this fully plastic scenario, the contact pressure equals the indentation hardness; consequently, the ratio of total normal load *L* (carried by all asperities) over the hardness *H* of the soft material results in the real contact area: $A_{r}=L/H$.

Fully plastic contact conditions can be reasonably assumed to occur in the metal forming context [19,20]. Nevertheless, such a model assumes a constant hardness value, which, albeit true for severely cold worked metal (because they essentially do not work harden), is not appropriate depending on the situation. In the case of work-hardening metals, Tabor writes about using a “representative strain”, which depends on the geometry of the indenter, to evaluate the flow stress and maintain the proportionality between hardness and yield stress at a value of c≈3. With regards to temperature and strain rate effects, there seems to be no experimental agreement for the real dependence of hardness with the former [21] or extensive investigation on the effects of the latter. Nonetheless, the relation $H=c\sigma_{y}$ with c=2.8to3 is frequently used in the metal forming tribology literature [22,23,24], implying that $H\left(\varepsilon ,\dot{\varepsilon },T\right)=c\sigma_{f}\left(\varepsilon ,\dot{\varepsilon },T\right)$ with c=2.8to3 is assumed.

The indentation hardness, or mean contact pressure, of thermo-viscoplastic materials is not readily available in the literature, especially when indented by non-standard geometries. Analytical solutions for normal contact of single asperities are possible under simplifying assumptions and geometries [25,26], which can be extended to develop multi-asperity contact models, such as the well-known elastic Greenwood–Williamson model [27]. Nevertheless, for complex situations such as those of metal-forming operations, numerical methods such as the Finite Element (FE) Method are better suited to deal with contact problems that would otherwise be very complicated or impossible to solve analytically [28,29,30,31,32,33]. The use of numerical methods assist in the development of new tribological models that may include features presently lacking further efforts, for example, the inclusion of morphology and plasticity [2]. Recently, Shisode et al. [34] proposed an approach consisting of performing FE analyses to calculate contact pressure in the flattening of multiple coated asperities. In this manner, the total force carried by each asperity is obtained from a database of FE simulations. Along with further assumptions, a contact model was developed in a more customised and presumably more accurate way than simply assuming asperity tips always support 2.8 to 3 times the flow stress.

The approach of [34] inspired the development of this work. Here, instead of *flattening* of coated substrate, the FE models are *indentations* of asperities on a thermo-viscoplastic material. In this manner, Tabor’s concept of hardness as an average pressure is revisited but with the flexibility of allowing complex plastic flow not only due to different sizes and shapes of “indenters” but also due to the nonlinear interplay between strain, strain rate, and temperature and to the displacement of non-contacting regions evidenced by the sink-in and pile-up of material around the contact. The main goal is to investigate how the thermo-viscoplastic flow stress nature of a deformable body influences the load–area relation of the contact while building an appropriate contact model for a metal-forming situation. To achieve this goal, a metal-forming situation is analysed in which a thermo-viscoplastic smooth surface (representing a workpiece) is indented by a rough surface (representing a tool). The choice for a rough tool was motivated by the fact that rolled products are often imprinted and thus defined by the topography of the roll [11]. The workpiece material represents a 6061 aluminium alloy, for which multiple accurate constitutive relations are available. The rough surface of the tool is represented in such a way to consider coalescence of contact and formation of specific geometries of contact patches, which are treated as indenting asperities of different shapes in the FE model. In this sense, the hardness due to an indentation of a single asperity is viewed as a contact pressure derived from an FE simulation for that particular asperity. The database of FE simulations along with a surface representation algorithm allow for the development of a thermo-viscoplastic contact model.

## 2. Materials and Methods

### 2.1. Topography and Representation

A mill roll surface is commonly made of high strength steel and may range from mirror bright to “mill finish” depending on the operation and product to be manufactured [35]. Certain manufacturing processes, such as laser texturing, may result in a more deterministic pattern, whereas others, such as electrical discharge texturing, result in a random topography [36,37,38]. In this work, an artificially created random isotropic surface, which can be generated following, e.g., [39,40], was used as the reference topography to represent a rigid rough tool. While it is true that surfaces are *rough* at different scales, the study presented here dealt with roughness in the micrometer range and, thus, in the “microtribology regime” as per [41].

**Figure 1:**
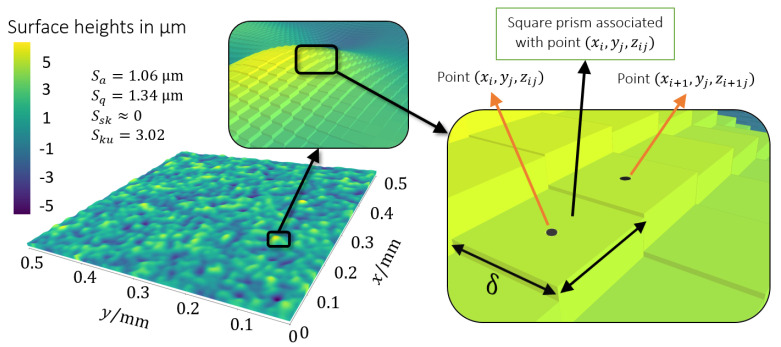
Artificial isotropic surface used as reference for this study; surface parameters such as arithmetical mean height ($S_{a}$), root mean square deviation ($S_{q}$), skewness ($S_{sk}$), and kurtosis ($S_{ku}$) are displayed. Surface points and associated square prisms representing the surface are detailed.

The generated surface and characterising surface parameters are shown in a Cartesian coordinate system in Figure 1; it was generated from a set of 501 × 501 surface points ($x_{i},y_{j},z_{ij}$) such that $x_{i}=i*\Delta x$ and $y_{j}=j*\Delta y$ form a regular grid (Δx=Δy=1 μm) on the mean plane and $z_{ij}$ is the signed distance normal to that plane. Between these points, the surface is approximated using *nearest-neighbour* interpolation (the value at any location is equal to the value of the nearest surface point, resulting in a piecewise-constant representation), which creates a second grid consisting of square pixels of side length δ (with δ=Δx=Δy=1 μm), each associated with (and centered at) a surface point. Therefore, the surface is in fact represented by a collection of *square prisms*, i.e., two dimensional square pixels of side length δ with an offset $z_{ij}$, which is called the surface height and defines the height of the prism. One may note that the square pixels associated with the surface points at the edge of the surface creates an additional area at the border of the surface. Nonetheless, the nominal area considered for analysing the results was kept at $A_{n}=0.5\mathrm{mm}\times 0.5\mathrm{mm}=0.25{\mathrm{mm}}^{2}$ since that is the original intended size of the generated surface.

In contacts involving a significant degree of contact, it is not realistic to assume isolated asperity contacts and so, the phenomenon of *asperity coalescence* should be taken into account. As Greenwood writes, the number of contacts do not simply increase as surfaces approach each other, but they rather merge or coalesce and form “contact patches” [42]. In order to account for coalescence and the formation of contact patches, a deterministic approach considering coalescence of surface heights is used in this work following that described by Ma et al. [20]. Basically, the surface is analyzed by an algorithm, which firstly computes the distance between $z_{ij}$ and a hypothetical plane at certain fixed separation *s* from the mean plane of the surface. The hypothetical plane represents a perfectly smooth solid body, thus if $z_{ij}\geq s$, then $z_{ij}$ is considered to be in contact. Next, all contacting surface heights (each associated with a square prism) are grouped in clusters by recognizing which $z_{ij}$ are neighbours to each other. By neighbours, a two dimensional 4-connected sense is denoted, which means that neighbouring heights share at least an edge in their square base [43]. A connected cluster of surface heights forms what is called a contact patch, which in general will have a complex and unique shape.

**Figure 2:**
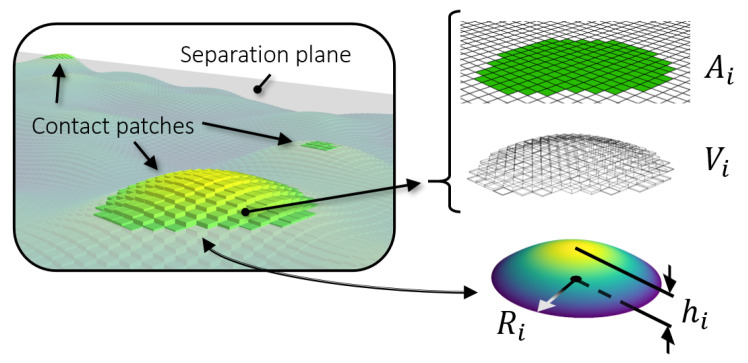
Identification and representation of a contact patch using the patch area ($A_{i}$) and volume ($V_{i}$) to build a quadratic surface of the same area and volume defined by a radius ($R_{i}$) and height ($h_{i}$).

Once all the contact patches are identified, they are represented by simple quadratic surfaces. For such purpose, the surface algorithm evaluates the base area $A_{i}$ of the contact patch (the sum of all square pixels area belonging to that patch) and the volume $V_{i}$ of the contact patch (the sum of the volume of all square prisms belonging to that patch), as illustrated in Figure 2. The volume of a single square prism is the base area $\delta^{2}$ times the height ($z_{ij}-s$). After $A_{i}$ and $V_{i}$ are computed, the contact patch is represented in this work by a *circular paraboloid* [44]. Using $A_{i}$ and $V_{i}$, the base radius $R_{i}$ and height $h_{i}$ of the paraboloid can be obtained according to Equations (1) and (2)

(1)Ai=niδ2→Ri=Aiπ(2)Vi=∑δ2(zij−s)   forzij∈contact patch i→hi = 2ViπRi2

where $n_{i}$ is the number of surface heights of the patch. In other words, the paraboloid base radius is calculated equating the patch area to that of a circle while the height of the paraboloid is calculated equating the patch volume to that of a circular paraboloid. More accurately, the patches could be represented using elliptical paraboloids or ellipsoids [45]. The reason for choosing paraboloids with equal major and minor axes, i.e., circular paraboloids, was to limit the space domain of the FE model to two-dimensional axisymmetric. Elliptical paraboloids would require a three-dimensional FE model for a faithful representation, which was not explored in this work. Furthermore, while asperities of anisotropic surfaces are not of axisymmetric shape, those of isotropic surfaces can be reasonably represented by such an approximation. Alternative to the circular paraboloid, the contact patches could also be represented by other axisymmetric quadratic surfaces, such as the *spheroid* (or ellipsoid of revolution), as used by [34]. An immediate difference between paraboloids and (half) spheroids is the different height for the same volume and base radius: the circular paraboloid height is calculated as $2V_{i}/\pi R_{i}^{2}$, whereas the height of the half spheroid would be $3V_{i}/2\pi R_{i}^{2}$, which is 0.75 times that of the paraboloid for the same base radius and volume.

### 2.2. Degree of Separation

**Figure 3:**
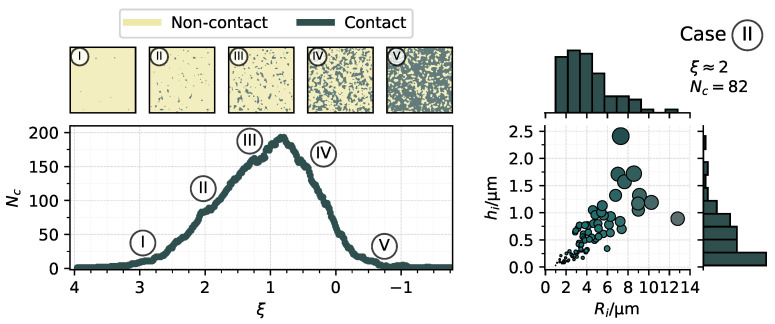
Left: Number of contact patches $N_{c}$ according to dimensionless separation ξ and snapshots of the contact interface at selected separations. Right: Distribution of contact patches (marker size scaled by patch area) in terms of radii and heights for case II, along with marginal histograms.

The number of surface heights in contact progressively increases as the separation decreases. The number of contact patches also initially increases and grows, but as the patches become increasingly connected, the total number starts to decrease. This evolution along a finite number of separations is shown in Figure 3 in terms of a dimensionless separation ξ, i.e., the separation *s* normalised by the root mean square deviation of the surface $S_{q}$. The images on top show in dark color the evolution of surface heights in contact for select positions. On the right of the figure, the distribution of contact patches for case 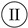 is detailed.

Generally, tribological aspects of a contact are associated with events on the surface, but deformation of the underlying bulk material may play an important role on the contact. In the context of metalworking tribology, it is well known that, in operations such as rolling, sub-surface bulk deformation causes asperities to flatten more due to a decrease in the effective hardness [46,47], which consequently causes the contact area to increase. For this reason, the evolution shown in Figure 3 should be viewed with care. In this work, the effect of the bulk is not explored and, therefore, the methodology discussed previously can be seen as a way to study the initial moments of a metal-forming contact before bulk deformation significantly affects the contact. Furthermore, since the focus of the present investigation was on the influence of the material properties, only a single separation was selected for the study, namely at ξ≈2, which resulted in the situation of case 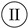, detailed in Figure 3. At such a separation, a noticeable number of diversely shaped contact patches are resolved and it is reasonably assumed that other effects, such as the bulk deformation, or the presence of a lubricant did not play a major role in the contact yet.

### 2.3. Single Asperity Finite Element Model

**Figure 4:**
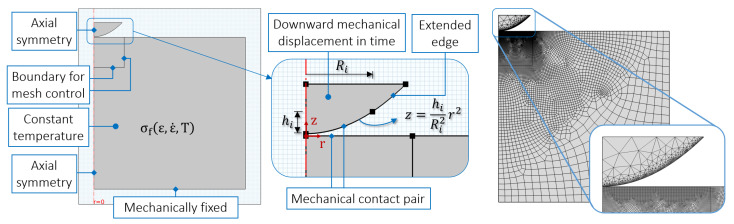
Reference FE model with indicated boundary and domain conditions. Typical mesh of a simulation is shown on the right.

The context of the simulations is seen as the initial contact of a hot rolling process in the sense that asperities of a comparatively much harder rough surface (representing the roll) indents a hot workpiece at a certain speed. The roll material normally ranges from cast irons to high carbon steels [48] depending on the operation; consequently, they have much higher Young’s modulus and yield strength than aluminium, which justifies treating the asperities as rigid in the FE model. The database is built from a series of single asperity simulations; the “reference model” shown in Figure 4 considers only a normal approach, which can be justified by the limited indentation depths and small relative tangential motion in comparison to the normal approach between a tool and workpiece. Although heat transfer is a characteristic aspect of the actual process [49], it was not considered in the model; by treating the model as isothermal, one can analyse the contact results in terms of thermo-viscoplastic deformation response of the material alone and not a combination of material model and heat transfer coefficients. Otherwise, the contact results would depend on an asperity-dependent heat flow, which would prevent a direct comparison between different sized asperities.

The commercial software COMSOL Multiphysics^®^ v. 5.2a was used to perform the modeling and simulations [50]. A two-dimensional axisymmetric space and a time dependent study were set. There are two domains in the model: the contact patch (or asperity) of the roll defined by its base radius and height (domain detailed in the middle of Figure 4) and the rectangular shaped substrate representing the aluminium workpiece. The asperity is modelled with an extended edge to account for the effects of the material surrounding the contact patch in the indentation; this allows to study whether this region is contacted by the deforming substrate and how the contact area deviates from that of the surface algorithm (more details in Section 3). Structurally, the substrate bottom boundary is fixed and the asperity moves downwards during a certain time, which is equivalent to specifying a velocity. The movement is prescribed to the domain so that the asperity behaves rigidly throughout the transient study without consideration of inertial terms; a penalty formulation is used for the frictionless contact.

### 2.4. Material Models

The material model of the workpiece studied in this work was that of the 6061 aluminium alloy, a precipitation-hardenable alloy containing Mg and Si as the main alloying elements, which is widely used for structural shapes and commonly manufactured by hot rolling. In a previous work [15], different constitutive relation functions of plastic strain, plastic strain rate, and temperature were constructed and compared for flow curves of this alloy in as-cast conditions, i.e., a 6061-F aluminium alloy. Here, those material models presenting the best results in terms of accuracy with the experimental data were considered, namely the Garofalo–Arrhenius (GA), the “new Johnson–Cook” (nJC), and the Hensel–Spittel (HS) models, with the GA model in particular showing the best results.

The flow stress predicted by the GA model is given by

(3) $$\sigma_{GA}\left(\varepsilon ,\dot{\varepsilon },T\right)=\frac{1}{\alpha (\varepsilon )}\mathrm{arcsinh}\left[{\left(\frac{\dot{\varepsilon }}{s^{-1}}\frac{\exp\left(Q(\varepsilon )/RT\right)}{\exp\left[\ln\left(A_{GA}\left(\varepsilon \right)/s^{-1}\right)\right]}\right)}^{\left(1/n^{{}^{\prime }}\left(\varepsilon \right)\right)}\right]$$

where R=8.314 J/mol/K and the material “constants” $A_{GA}\left(\varepsilon \right),\alpha \left(\varepsilon \right),n^{{}^{\prime }}\left(\varepsilon \right)$, and Q(ε) are higher-order polynomial functions of the equivalent plastic strain ε (see Appendix A).

The HS model is commonly written as

(4) $$\sigma_{HS}\left(\varepsilon ,\dot{\varepsilon },T\right)=A_{HS}e^{m_{1}T}\varepsilon^{m_{2}}e^{m_{4}/\varepsilon }{\left(1+\varepsilon \right)}^{m_{5}T}e^{m_{7}\varepsilon }{\left(\dot{\varepsilon }/s^{-1}\right)}^{\left(m_{3}+m_{8}T\right)}$$

where $A_{HS},m_{1},m_{2},m_{3},m_{4},m_{5},m_{7}$ and $m_{8}$ are material constants given by Table 1.

Finally, the nJC model flow stress is written as

(5) $$\sigma_{nJC}\left(\varepsilon ,\dot{\varepsilon },T\right)=\left[A_{nJC}+B\varepsilon^{n}\right]\left[1+C\ln\left({\dot{\varepsilon }}^{*}\right)\right]\exp\left[\left(\lambda_{1}+\lambda_{2}\ln\left({\dot{\varepsilon }}^{*}\right)\right)\left(T-T_{ref}\right)\right]$$

where $A_{nJC},n,B,C,\lambda_{1}$, and $\lambda_{2}$ are material constants given in Table 2. The term ${\dot{\varepsilon }}^{*}=\dot{\varepsilon }/{\dot{\varepsilon }}_{0}$ is a dimensionless strain rate, where ${\dot{\varepsilon }}_{0}=0.1\;s^{-1}$ is a reference strain rate, $\dot{\varepsilon }$ is the current strain rate, *T* is the deformation temperature, and $T_{ref}=400{}^{\circ}C$ is a reference deformation temperature.

**Figure 5:**
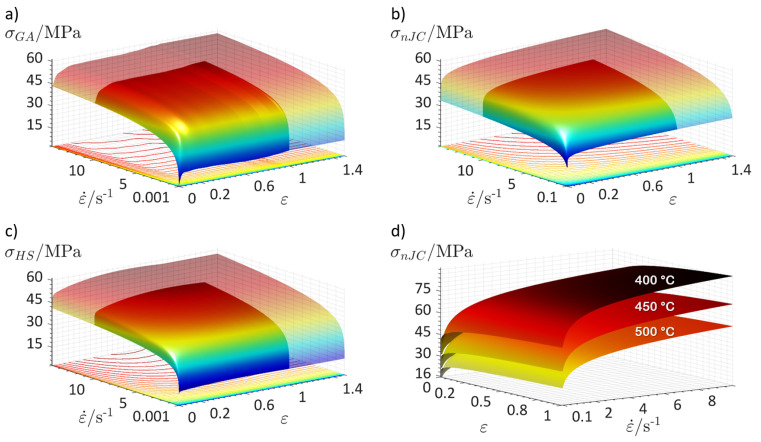
Flow stress predicted by the (**a**) GA model, (**b**) nJC model, and (**c**) HS model as a function of plastic strain and plastic strain rate at a temperature of 500°C; (**d**) changes in the nJC flow stress levels due to different temperatures.

For visualisation purposes, the material models are plotted as surfaces in Figure 5, where the changes in flow stress with plastic strain and plastic strain rate at a temperature of 500 °C can be visualised. The temperature have essentially the same effects on the material models, which is shown using the nJC model as an example on the bottom right image of Figure 5; the general shape of the surface remains the same, but its range of stresses is shifted upwards for lower temperatures and downwards for higher. Since each model has its own mathematical formulation, they present slightly different predictions of flow stress. The nJC model, for instance, due to its own formulation, considers strain rate effects only for $\dot{\varepsilon }\geq 0.1\;s^{-1}$; for $\dot{\varepsilon }<0.1\;s^{-1}$, the flow stress is extrapolated to have the same value as that at $\dot{\varepsilon }=0.1\;s^{-1}$ (while still being a function of ε and *T*). On the other hand, the GA and the HS capture strain rate effects for $\dot{\varepsilon }\geq 0.001\;s^{-1}$. The translucent regions in the figure refer to predictions lying outside the range of the experimental data, namely, for ε>1 and $\dot{\varepsilon }>10\;s^{-1}$. At these regions, the flow stress parameters ε and $\dot{\varepsilon }$ were set to remain constant for the GA and HS models in order to avoid unrealistic flow stress values resulting from these models. For the nJC, extrapolation did not reveal any anomaly in the predicted stress, and thus, no extrapolation correction was performed.

The material models were implemented in the FE model by writing the hardening law of the material as a user-defined analytic function. The material dependency on time, i.e., the strain rate variable, was taken into account since the simulations were time-dependent. The large plastic deformation option was used, which means that plasticity was based on the multiplicative decomposition (elastic and plastic) of the deformation gradient. In reality, the model was elastoplastic; the linear elastic properties of the substrate were set as 70 GPa for Young’s modulus and 0.33 for Poisson’s ratio, which are standard values for aluminium. Nonetheless, the flow stress was reached nearly as soon as contact was established, rendering the elastic properties irrelevant. For plasticity, the distortion-energy theory, or von Mises yield criterion was used, i.e., plastic deformation occurs when $\sigma_{VM}\geq \sigma_{y}$, where $\sigma_{VM}$ (defined as a single, effective, or equivalent stress, called *von Mises stress*) is a scalar value computed from the Cauchy stress tensor written, in its general format, as $\sigma_{VM}^{2}=0.5\left[{\left(\sigma_{11}-\sigma_{22}\right)}^{2}+{\left(\sigma_{22}-\sigma_{33}\right)}^{2}+{\left(\sigma_{33}-\sigma_{11}\right)}^{2}+6\left(\sigma_{23}^{2}+\sigma_{31}^{2}+\sigma_{12}^{2}\right)\right]$. The problem was highly nonlinear not only due to the material model but also due to the contact formulation itself, which implies in geometric nonlinearity [51].

## 3. Results and Discussion

The FE simulations allowed us to build a database from which the influence of the thermo-viscoplastic behaviour of the deforming material in the load–area relation was investigated. The results of the FE simulations are analysed from a single asperity perspective and from the rough surface perspective, i.e., the toll–workpiece contact. In order to investigate how strain, strain rate, and temperature affect the load–area relation of the contact, the indenting velocity of the asperities and the temperature of the substrate were varied. The effects from changing the material model was also investigated. The analysis is focused on four different aspects of the contact evaluated from the results database: the contact load, the average pressure supported by the substrate, the real contact area, and the displacement of the non-contacting area.

### 3.1. Asperity

#### 3.1.1. Strain, Strain Rate, and Temperature

A single indenting asperity is analysed under velocities of 0.01 μm/s, 0.1 μm/s, and 1 μm/s, combined with temperatures of 400°C, 450°C, and 500°C. The strain and strain rate effects are evidenced by changes in the indenting velocity, since it modifies strain rate fields in the deforming material and, consequently, how the deformation develops. Similarly, temperature effects on the material are reflected by changes in the substrate domain temperature. The asperity with the biggest $h_{i}$ is chosen since its indentation depth allows for an extensive visualisation of the effects of the variables. This “asperity *k*” has height $h_{k}=2.41\;\mu m$ and radius $R_{k}=7.29\;\mu m$.

**Figure 6:**
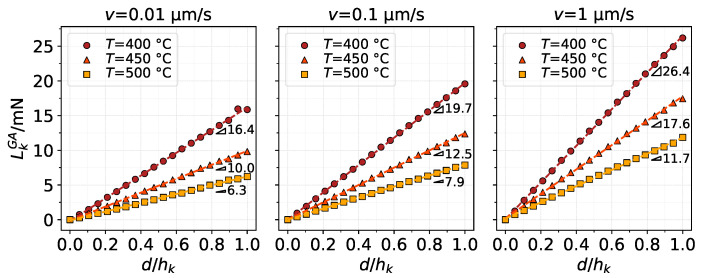
Contact load evaluated from the FE model as asperity *k* indents the substrate using the GA material model. Results for different conditions of indenting velocity and substrate temperature.

Figure 6 shows the vertical contact force $L_{k}^{GA}$ using the GA model. The contact load is obtained as the z-component of the total contact force from the FE simulation and is shown as a function of its indentation depth *d* normalised by $h_{k}$. Thus, $d/h_{k}=1$ means the asperity has penetrated a distance equivalent to its height $h_{k}$ into the substrate as measured from the original substrate surface level. The results show how the increase in temperature (causing softening of the substrate material) leads to a smaller load carrying capacity for the same indentation depths. Analogously, increasing the indenting velocity results in strain rate hardening of the material, leading to higher contact loads. Interestingly, despite the nonlinear nature of the material model, the load–indentation relation throughout the height of the asperity exhibits a nearly linear behaviour, which is quantified by the slope triangles in the figure displaying the rate at which the load increases per unit indentation depth in each case.

**Figure 7:**
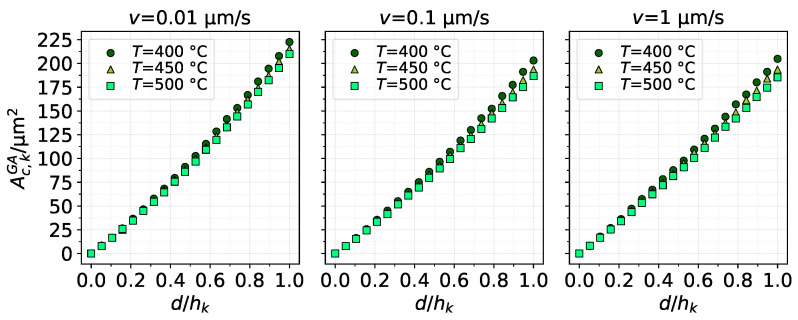
Contact area evaluated from the FE model as asperity *k* indents the substrate using the GA material model. Results for different conditions of indenting velocity and substrate temperature.

**Figure 8:**
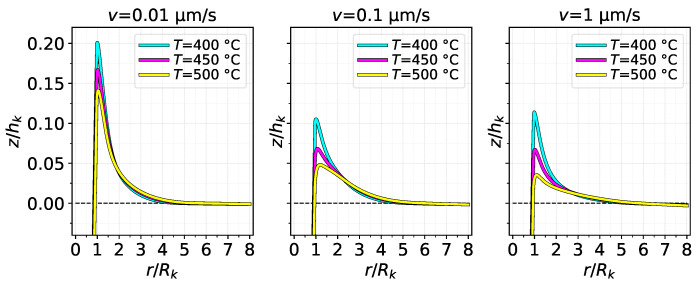
Surface profile of substrate along the normalized radius after full indentation of asperity *k*.

Figure 7 shows the contact area as a function of $d/h_{k}$ for the same cases as Figure 6. The contact area is calculated by integrating a Boolean equation on the contact pressure from the FE results. The area–indentation relation could also be reasonably modelled in a linear manner, but a slight quadratic behaviour is more evident. In general, all cases present similar values of contact area, but higher temperatures yielded smaller contact area at the end of indentation in all cases. An increase in the indenting velocity also seems to indicate a slight decrease in the contact area. It is worth highlighting the reason behind such a result, which has to do with piling-up of the material around the indentation. Figure 8 shows the surface profile of the substrate along the distance from the center of the indentation, normalised by $h_{k}$ and $R_{k}$, respectively. The profile is shown for $d/h_{k}=1$, i.e., at full indentation. Visibly, a higher pile-up occurs at the lowest indenting velocity, which consequently creates a higher probability that the surrounding material will contact the extended edge of the asperity (detailed in Figure 4), resulting, thus, in more contact area. On the other hand, a softer substrate (higher temperature) results in less pile-up, which lessens the contact area.

Figure 8 also suggests that strain hardening and temperature affect pile-up more than strain rate hardening. A increase in the indenting velocity did not increase the pile-up; in fact, it even decreased for the T=500 °C between v=0.1 μm/s and v=1 μm/s. Meanwhile, in the v=0.01 μm/s and T=400 °C case, the highest pile-up was observed at a nearly 20% rise relative to $h_{k}$. Interestingly, despite the varied heights and shapes of the piled-up surface, the material returns to the original level at practically the same position in all cases, which is at a distance of about $5R_{k}$; this may suggest that such a distance depends only on the geometry of the indenting asperity, although the general pile-up profile clearly depends on the thermo-viscoplastic conditions. It can also be noticed that, for $r/R_{k}⪆7$, the substrate undergoes a small reduction in height in some cases, which is linked to the lack of mechanical constraint for lateral displacement in the FE model.

**Figure 9:**
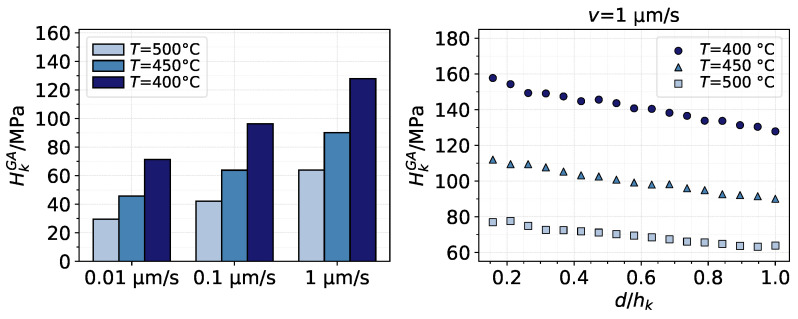
Left: Average contact pressure after full indentation of asperity *k* using the GA model. Right: decrease in average pressure as asperity *k* indents the substrate for v=1 μm/s using the GA model.

An evaluation of the average contact pressure (contact load over contact area) at full indentation, $H_{k}$, also exposes the effects of $\dot{\varepsilon }$ and *T*, as shown in the bar graph of Figure 9. The graph on the right of the figure shows that, in reality, $H_{k}$ decreases its value throughout the indentation (after quickly reaching its highest value at the start of the contact), which means that the contact area increases faster than the contact load. Thus, while the load caused by the indenting asperity continuously increases with depth, the deformation of the substrate material causes the average pressure to decrease. The tendencies shown in the figure were also observed to a lesser extent for other indenting velocities.

#### 3.1.2. Material Model

**Figure 10:**
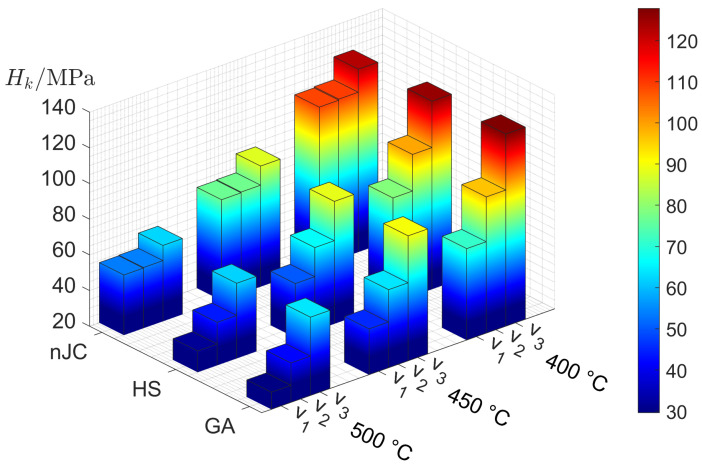
Average contact pressure after full indentation of asperity *k* using indicated material models, substrate temperature and indenting velocities: $v_{1}=0.01\;\mu m/s,v_{2}=0.1\;\mu m/s$ and $v_{3}=1\;\mu m/s$.

The previous analyses were also performed using the nJC and HS material models. The results are compared in terms of average pressure in Figure 10.

**Figure 11:**
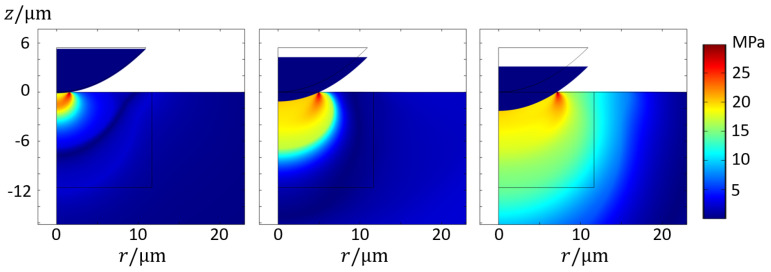
Stress field (*von Mises*) at different indentation time steps (v=0.01 μm/s and T=500 °C) using the GA model.

As with the GA model, the overall increase in average pressure with decreasing temperature and increasing indenting speed is also observed in the HS model. On the other hand, the nJC model practically does not show differences between $v_{1}$ and $v_{2}$. The reason lies in the fact that, at such indenting speeds, the range of strain rates in the substrate are generally below the nJC model’s $0.1\;s^{-1}$ threshold for consideration in the flow stress. Hence, from the point of view of the nJC model, $v_{1}$ and $v_{2}$ are practically the same. Additionally, since the nJC model considers that $\sigma_{nJC}\left(\varepsilon ,\dot{\varepsilon },T\right)=\sigma_{nJC}\left(\varepsilon ,0.1,T\right)$ for $\dot{\varepsilon }<=0.1\;s^{-1}$, there is an overestimation of $H_{k}$ for $v_{1}$ and $v_{2}$ in comparison to the other models. This result reveals how the correct description of the flow stress at small values of strain and strain rate can have significant influence in the development of the strain field and, thus, contact pressure caused by an indenting asperity. A visual inspection of the von Mises stress fields, as shown in Figure 11, show the complex stress field in such an indentation of the thermo-viscoplastic material. During this process, the highest stresses (and also highest plastic strain and strain rates) always occurred at the most recent contact location, with the field propagating towards the interior of the substrate at lower values.

Another interesting aspect is to evaluate the pile-up/sink-in behaviour in each case through the contact area. From the surface algorithm of Section 2, the base area of an asperity *i* was obtained as $A_{i}$, which was then used to find $R_{i}$. With the volume $V_{i}$, the height $h_{i}$ was defined. A circular paraboloid defined by $R_{i}$ and $h_{i}$ results in a surface area that can be calculated by revolving its parabolic profile and by calculating the surface of revolution. The resulting expression for the surface area $A_{c,i}$ (not including the circular base) is given by the following:

(6) $$A_{c,i}=\frac{\pi R_{i}}{6h_{i}^{2}}\left[{\left(R_{i}^{2}+4h_{i}^{2}\right)}^{3/2}-R_{i}^{3}\right]$$

**Figure 12:**
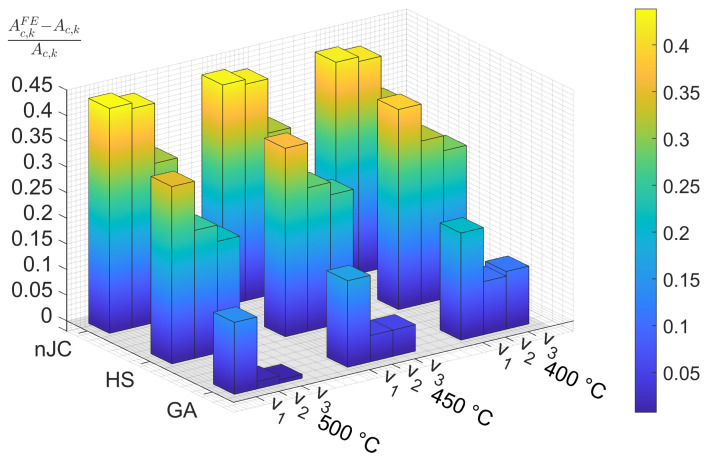
Relative difference of $A_{c,k}^{FE}$ and $A_{c,k}$ after full indentation of asperity *k* using indicated material models, substrate temperature and indenting velocities: $v_{1}=0.01\;\mu m/s,v_{2}=0.1\;\mu m/s$ and $v_{3}=1\;\mu m/s$.

In the FE model, pile-up and/or sink-in of the substrate material surrounding the indentation, which is not taken into account in the surface algorithm, may cause the contact area of asperity *i* from the FE simulation, $A_{c,i}^{FE}$, to deviate from $A_{c,i}$ (calculated according to Equation (6)). Since the geometry of the asperity in the FE model is built with an extended edge (see Figure 4), pile-up of the substrate material is likely to contact the asperity at that region, which is identified by comparing $A_{c,i}^{FE}$ to $A_{c,i}$. Figure 12 compares the relative difference of the contact area in the indentation of asperity *k* using the nJC, HS, and GA models.

The GA model results in very small differences, whereas the nJC and HS models result in contact areas clearly larger than that calculated from the surface algorithm, which means a general piling-up of the substrate occurs and contacts the asperity at the extended edge region lying above the original level of the substrate surface. As with the average pressures, the nJC model practically shows the same results for $v_{1}$ and $v_{2}$, and values greater than the HS and GA models. The reasons can be again attributed to the formulation of the nJC model, as previously discussed for the average pressure. The similarity betwen the results for the HS and GA models in the average pressure does not repeat in Figure 12, where evident differences are visible; this shows how slightly different mathematical descriptions of the material model may result in significantly different deformation patterns. It was also verified that the size of the FE mesh had a significant effect on the contact area at the near-edge region of the contact, since the deformation is inherently linked to the size of the FE elements; a mesh convergence was performed to ensure that the results are minimally affected by the size of the mesh.

**Figure 13:**
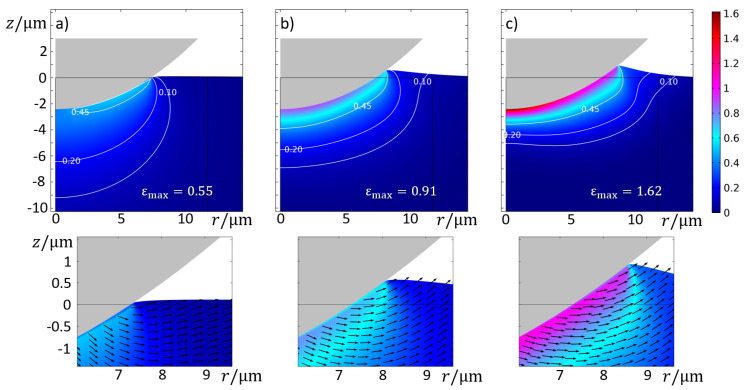
Equivalent plastic strain field and contour after full indentation of asperity *k* using the (**a**) GA model, (**b**) HS model, and (**c**) nJC model for v=0.01 μm/s and T=500 °C; bottom images detail the contact edge region with an arrow field of the displacement.

**Figure 14:**
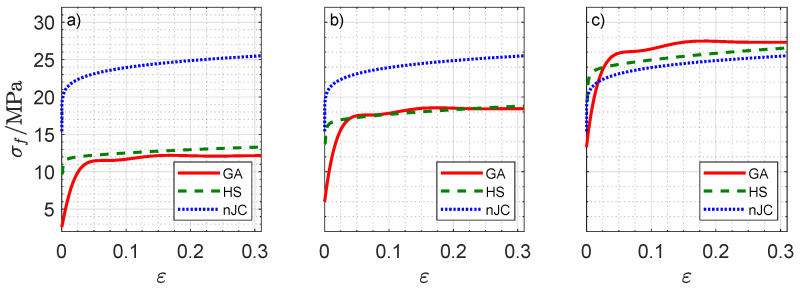
Flow stress predictions according to the GA, HS, and nJC models at strain rates of (**a**) $\dot{\varepsilon }=0.001\;s^{-1}$, (**b**) $\dot{\varepsilon }=0.01\;s^{-1}$, and (**c**) $\dot{\varepsilon }=0.1\;s^{-1}$ as a function of plastic strain at a temperature of 500 °C.

Figure 13 details the complex material flow creating the differences in Figure 12. The plastic strain fields show that the maximum equivalent plastic strain $\varepsilon_{max}$ for the GA model was the smallest among the three models but that the strain field is more spread out, as evidenced by the contour of the plastic regions. In the nJC case, the plastic strains are more concentrated, which is likely because of the lack of strain rate hardening for $\dot{\varepsilon }<0.1\;s^{-1}$, causing the substrate to initially accumulate the plastic strain before the stress can propagate throughout the material. An evaluation of the solution fields throughout the indentation revealed that strain rate ranges from 0 to $0.064\;s^{-1}$ for the GA model, $0.096\;s^{-1}$ for the HS model, and $0.159\;s^{-1}$ for the nJC model. It is important to recall that the material models result in noticeably different predictions at small strain and strain rate values, as detailed in Figure 14. Clearly, the pronounced strain hardening at the beginning of the flow curves occurs at a relative wide range of strains (up to ε≈0.05) for the GA model, whereas the HS and nJC models reach a nearly constant value in a much smaller range. The strain and stress fields in the initial moments of a deformation process greatly define the subsequent deformation of the body. In this sense, different predictions at small strains and strain rates are believed to be the main cause of different FE results in Figure 13. Evidently, such results are a consequence not only of the material model but also of the asperity geometry and type of mechanical constraint of the substrate.

### 3.2. Surface

**Figure 15:**
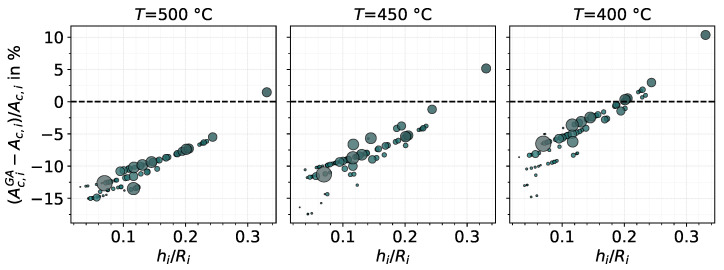
Relative difference (in percentage) of $A_{c,i}^{GA}$ and $A_{c,i}$ as a function of the aspect ratio ($h_{i}/R_{i}$) of all asperities after full indentation using the GA model (marker size scaled by $A_{c,i}$); indenting velocity of v=0.1 μm/s and substrate temperature as indicated.

In this section, we investigate the total load–area relation of the selected separation in Section 2.2, i.e., considering the $N_{c}=82$ asperities of case 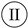 (Figure 3). For this goal, the database of the GA model is used. As discussed previously, $A_{c,i}^{FE}$ may deviate from $A_{c,i}$ consequently causing the total contact area from the FE database to deviate from that calculated by the surface algorithm. In Figure 15, the database contact area of each asperity, $A_{c,i}^{GA}$, is compared to that given by Equation (6), $A_{c,i}$ in terms of a relative difference plotted against the aspect ratio $h_{i}/R_{i}$.

The figure shows that $A_{c,i}^{GA}$ is generally smaller than that predicted by Equation (6). The rightmost marker in the figure refers to asperity *k*, which evidently is not a representative behaviour of the asperities of the surface; asperities were more likely to cause sink-in of the material surrounding the indentation. As the substrate temperature decreases and the material becomes harder, the sink-in make room for pile-up, subsequently reducing the difference but likely leading to a positive relative difference with further decrease in temperature. The distribution in the figure reveals an approximate quadratic tendency in the relative difference of the contact area with respect to the aspect ratio $h_{i}/R_{i}$, meaning that asperities of higher aspect ratios were less likely to cause sink-in. In a rather general way, asperities with bigger contact area (displayed by the marker size) were also less likely to sink-in in comparison to asperities of smaller areas.

**Figure 16:**
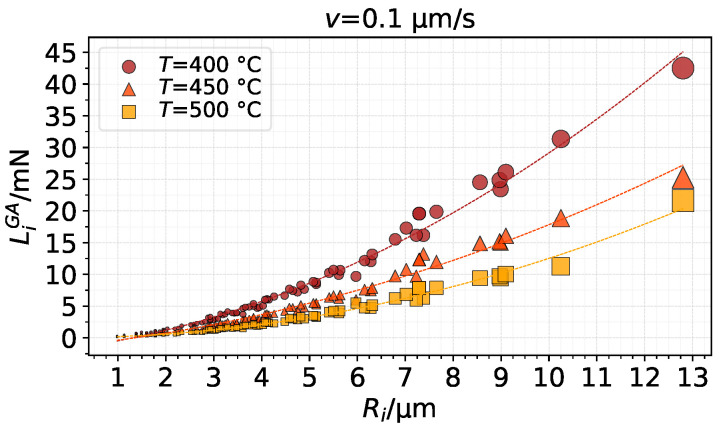
Contact load as a function of asperity radius after full indentation using the GA model (marker size scaled by $A_{c,i}$).

With regards to contact load, a nearly quadratic relation was found between contact load and radii, as shown in Figure 16. As expected, bigger asperities carry higher contact loads.

**Figure 17:**
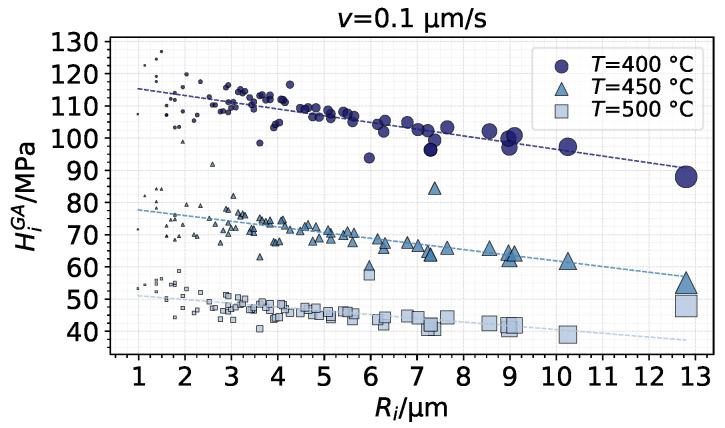
Average pressure as a function of asperity radius after full indentation using the GA model (marker size scaled by $A_{c,i}$).

In terms of average pressure, the distribution also as a function of the radii is shown in Figure 17. Visibly, the values of average pressure, or hardness, show a decreasing tendency with increasing radius and area, with the effects being more pronounced with colder and, thus, harder substrate. The results suggest that, in general, smaller asperities carry more pressure than larger ones in such a thermo-viscoplastic material.

Table 3 sums up the overall results in terms of total contact load and area, which can be said to be the results of the thermo-viscoplastic contact model. The superscript GA is omitted from the total variables for improved readability. The total normal load *L* carried by contacting asperities between toll and workpiece, and the total contact area $A_{r}$ (or real contact area) can be calculated as the sum of the contribution of all asperities, i.e., $L=\sum_{i}^{N_{c}}L_{i}^{GA}$ and $A_{r}=\sum_{i=1}^{N_{c}}A_{c,i}^{GA}$. Table 3 also shows the total degree of contact ($A_{r}/A_{n}$) for different studied cases.

It is important to highlight that the values of Table 3 assume that a superposition of the load and area of each asperity is valid for calculation of the total load *L* and total contact area $A_{r}$. The deterministic contact patch approach used (Section 2) indeed attempts to account for the interaction between surface heights, which was done by identifying connected surface heights and, thus, coalescence. Nevertheless, two separated contact patches may still be “close-enough” to each other in such a way that their stress fields may affect one another, consequently affecting the load they support and contact area. Such effects, explored for example by [52], were not investigated in this work.

**Figure 18:**
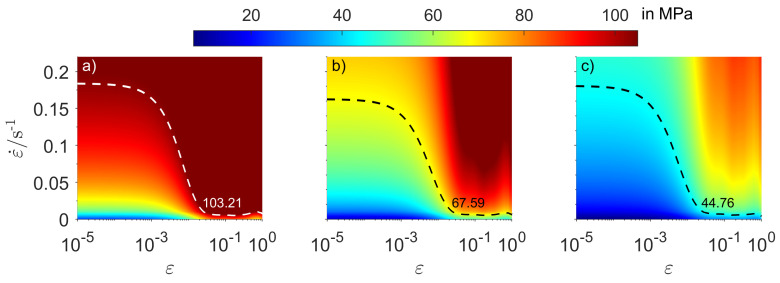
Surface plot of $2.8*\sigma_{GA}$ as a function of strain (in logarithmic scale) and strain rate (in linear scale) at temperatures of (a) 400 °C, (b) 450 °C and (c) 500 °C.

Finally, with the results of Table 3, a parallel to the fully plastic model can be drawn. If the contact model was developed using a fully plastic approach, i.e., a model such that $p_{n}A_{n}=A_{r}H_{eq}$, where $p_{n}$ is the nominal pressure between toll and workpiece, one may write $p_{n}A_{n}=L$ to calculate $H_{eq}=L/A_{r}$, which would be an equivalent hardness value to obtain the same results in terms of contact area and contact load of Table 3. It is interesting to verify how the values of $H_{eq}$ compare to the constraint factor times the flow stress of the material, since this is often used to express hardness of a material. For such purpose, Figure 18 shows $c\times \sigma_{GA}$ with c=2.8 for different values of strain and strain rate at the temperatures investigated. Dashed contour lines represent values equal to $H_{eq}$ according to Table 3.

Figure 18 reveals that, in order to obtain the same results as the thermo-viscoplastic contact model performed in this work, in terms of contact area and carried load, a fully plastic contact model in the format $H_{eq}=c\sigma_{f}\left(\varepsilon ,\dot{\varepsilon },T\right)$ should consider flow stress $\sigma_{f}$ evaluated at certain nonzero values of strain and strain rate, denoted by the contour line in Figure 18. Although the values of ε and $\dot{\varepsilon }$ are relatively small, the flow stress gradient at small strains and strain rates is evidently very pronounced for such materials, which causes the material to harden significantly fast. Consequently, the advantages of correctly describing the material behaviour at low strains and strain rates is evidenced again. Another remark to be made is that the displayed contour line is valid for a constraint factor of c=2.8 and for the separation studied in this section. If these conditions are changed, the value of $H_{eq}$ is also expected to change. Cases (a) and (b) show that the decrease in temperature (and thus hardening of the material) causes the contour line to move towards higher ε and $\dot{\varepsilon }$, which is also expected to occur similarly for higher indenting velocities. When the material shows less pronounced variations at low strains and strain rates, such as in the 500 °C case (c), the contour line may also move towards higher ε and $\dot{\varepsilon }$.

The approach using a FE database has the advantage of automatically resulting in an equivalent hardness through quantification of load and area while accurately and simultaneously considering the shape and sizes of the contacting asperities. The contact load quantification may be directly related to the external load through equilibrium conditions, while the contact area in combination with custom-shaped asperities allow for the development of physically based investigations on friction.

## 4. Conclusions

In this work, an approach to calculate contact mechanics quantities such as load and area of a contact involving the indentation of a temperature-dependent viscoplastic material was presented. The thermo-viscoplastic material was characterised by constitutive relations of a 6061 aluminium alloy, whereas the indenter was characterised by a circular paraboloid considering coalescence of surface heights. The effects of temperature, indenting velocity, material model, and asperity geometry were investigated by performing multiple FE analysis, allowing the following conclusions to be made:

- The thermo-viscoplastic parameters of the indented material had a clear influence on contact load, contact area, average pressure, and pile-up/sink-in. Despite the nonlinear nature of the material model, load and contact area of a single asperity showed a fairly linear behaviour with indentation depth whereas average pressure tended to decrease slightly after reaching a maximum at the start of contact.
- The movement of the initially non-contacting region, i.e., the sink-in/pile-up behaviour, affects the contact area computation and is significantly dependent on the thermo-viscoplastic conditions and choice of material model.
- Strain rate effects at very small strain rates significantly affected contact load and contact area calculation, as evidenced by the comparisons between different material models and particularly the nJC model. Overall, the choice of the material model had more pronounced effects on area computations than on contact load.
- Simulations of diversely shaped asperities showed a quadratic dependence of the contact load on the radius of the asperity. Nonetheless, asperities with smaller radii supported more pressure than asperities with bigger radii.
- The use of an equivalent hardness value for a fully plastic contact model should be obtained by evaluating the thermo-viscoplastic flow stress at nonzero values of strain and strain rate depending on the temperature.

## Figures and Tables

**Table 1:** Material constants for the HS model.

$A_{\mathrm{HS}}/$MPa	$m_{1}/K^{-1}$	$m_{2}$	$m_{3}$	$m_{4}$	$m_{5}/K^{-1}$	$m_{7}$	$m_{8}/K^{-1}$
5179.35	−0.006486	0.005376	−0.228525	−0.000186	0.001444	−0.668259	−0.000489

**Table 2:** Material constants for the nJC constitutive model.

$A_{\mathrm{nJC}}/$MPa	B/MPa	*n*	*C*	$\lambda_{1}/K^{-1}$	$\lambda_{2}/K^{-1}$
31.2161	24.3329	0.149543	0.140036	−0.00705089	0.000489547

**Table 3:** Total normal load (*L*), total contact area ($A_{r}$), degree of contact ($A_{r}/A_{n}$) and equivalent hardness ($H_{eq}$) for indenting velocity of v=0.1 μm/s.

	400 °C	450 °C	500 °C
$A_{r}/\mu m^{2}$	5688.71	5437.45	5293.03
L/mN	587.14	367.5	236.99
$A_{r}/A_{n}$	2.28%	2.17%	2.12%
$H_{eq}/\mathrm{MPa}$	103.21	67.59	44.76

## Data Availability

The data presented in this study are available upon request from the corresponding author.

## List of Symbols

The following abbreviations and symbols are used in this manuscript:

$A_{c,i}$	Surface area of general paraboloid-represented contact patch *i* (or simply
	“asperity *i*”)
$A_{c,k}$	Surface area of paraboloid-represented contact patch *k* (or simply “asperity *k*”)
$A_{c,i}^{FE},A_{c,k}^{FE}$	Contact area of asperity *i* and *k*, respectively, from FE simulation
$A_{c,i}^{GA},A_{c,k}^{GA}$	Contact area of asperity *i* and *k*, respectively, from FE simulation using the
	GA model
$A_{HS},m_{1},m_{2},m_{3},$	HS model material constants
$m_{4},m_{5},m_{7},m_{8}$	
$A_{i}$	Base area of contact patch *i*
$A_{n}$	Nominal contact area
$A_{nJC},n,B,C,$	nJC model material constants
$\lambda_{1},\lambda_{2}$	
$A_{r}$	Real contact area
*c*	Constraint factor
*d*	Indentation depth
DET	Abbreviation: Distortion-energy theory
FE	Abbreviation: Finite Element
GA	Abbreviation: Garofalo-Arrhenius
HS	Abbreviation: Hensel-Spittel
*H*	Indentation hardness
$H_{eq}$	Equivalent hardness
$h_{i}$	Height of general paraboloid-represented contact patch *i* (or simply “asperity *i*”)
$h_{k}$	Height of paraboloid-represented contact patch *k* (or simply “asperity *k*”)
$H_{k}$	Average contact pressure of asperity *k*

$H_{k}^{GA}$	Average contact pressure of asperity *k* from FE simulation using the GA model
*L*	Total normal load
$L_{k}^{GA}$	Vertical contact load caused by asperity *k* from FE simulation using the GA model
$N_{c}$	Number of contact patches at separation
$n_{i}$	Number of surface heights that belong to contact patch *i*
nJC	Abbreviation: new Johnson-Cook
$p_{n}$	Nominal pressure
*R*	Universal gas constant
$R_{i}$	Radius of general paraboloid-represented contact patch *i* (or simply “asperity *i*”)
$R_{k}$	Radius of paraboloid-represented contact patch *k* (or simply “asperity *k*”)
*s*	Surface separation
$S_{a}$	Arithmetical mean height
$S_{ku}$	Kurtosis
$S_{q}$	Root mean square height
$S_{sk}$	Skewness
*T*	Temperature
$T_{ref}$	Reference deformation temperature
*v*	Indentation velocity of asperity
$v_{1},v_{2},v_{3}$	Indentation velocities
$V_{i}$	Volume of contact patch *i*
$x_{i},y_{j},z_{ij}$	Surface points coordinates in Cartesian coordinates system
z,r	Spatial coordinates in axisymmetric coordinates system
$\alpha \left(\varepsilon \right),n^{{}^{\prime }}\left(\varepsilon \right),Q\left(\varepsilon \right),$	GA model material constants
$\ln\left[A\left(\varepsilon \right)/s^{-1}\right]$	
δ	Side length of square pixel and of square prism base
Δx,Δy	Distance between surface points in x−y plane
$\varepsilon ,\varepsilon_{max}$	Equivalent plastic strain and maximum equivalent palstic strain
$\dot{\varepsilon }$	Equivalent plastic strain rate
${\dot{\varepsilon }}^{*}$	Dimensionless strain rate for nJC model
${\dot{\varepsilon }}_{0}$	Reference strain rate for nJC model
$\sigma_{f},\sigma_{y},\sigma_{VM}$	Flow stress, yield stress, and von Mises stress
$\sigma_{GA},\sigma_{HS},\sigma_{nJC}$	Flow stress predicted by GA, HS, and nJC models, respectively
ξ	Dimensionless separation

## Appendix A

The constitutive model in the format that relates a hyperbolic sine and the Zener–Hollomon [53] parameter suggested by Sellars and McTegart [54] may also include a strain-compensated approach as proposed by Slooff et al. [55]. Such is the approach employed in previous work [15] and used in this work. Thus, the GA material constants $\alpha \left(\varepsilon \right),n^{{}^{\prime }}\left(\varepsilon \right),Q\left(\varepsilon \right)$, and $\ln\left[A\left(\varepsilon \right)/s^{-1}\right]$ are expressed as polynomial functions of the equivalent plastic strain ε as follows:

(A1) $$\begin{array}{cc} \alpha (\varepsilon ) & =C_{0}+C_{1}\varepsilon^{}+C_{2}\varepsilon^{2}+...+C_{p}\varepsilon^{p}\\ n^{{}^{\prime }}\left(\varepsilon \right) & =D_{0}+D_{1}\varepsilon^{}+D_{2}\varepsilon^{2}+...+D_{p}\varepsilon^{p}\\ Q(\varepsilon ) & =E_{0}+E_{1}\varepsilon^{}+E_{2}\varepsilon^{2}+...+E_{p}\varepsilon^{p}\\ \ln\left[A\left(\varepsilon \right)/s^{-1}\right] & =F_{0}+F_{1}\varepsilon^{}+F_{2}\varepsilon^{2}+...+F_{p}\varepsilon^{p} \end{array}$$

where $C_{i},D_{i},E_{i}$, and $F_{i}$ with *i* from 1 to the degree of the approximation *p* are the regression coefficients and the term with i=0 is the dependent variable intercept. Degrees of 14, 14, 12, and 12 were, respectively, used for $\alpha \left(\varepsilon \right),n^{{}^{\prime }}\left(\varepsilon \right),Q\left(\varepsilon \right)$, and $\ln\left[A\left(\varepsilon \right)/s^{-1}\right]$, with values as shown in Table A1.

**Table A1:** Polynomial coefficients for material constants of the GA model.

$\alpha \left(\varepsilon \right)/{\mathrm{MPa}}^{-1}$	$n^{{}^{\prime }}\left(\varepsilon \right)$	$Q\left(\varepsilon \right)/\left(\mathrm{kJ}\;\cdot \;{\mathrm{mol}}^{-1}\right)$	$\ln\left[A_{\mathrm{GA}}\left(\varepsilon \right)/s^{-1}\right]$
$C_{0}=5.124243153404677\times 10^{-2}$	$D_{0}=2.710628355015069$	$E_{0}=1.47305623421818\times 10^{2}$	$F_{0}=2.1448699217755347\times 10^{1}$
$C_{1}=-9.312859289097236\times 10^{-1}$	$D_{1}=1.5762677659367267\times 10^{2}$	$E_{1}=4.985296464634795\times 10^{3}$	$F_{1}=6.925030459894832\times 10^{2}$
$C_{2}=2.2585497235810287\times 10^{1}$	$D_{2}=-4.4886518733118555\times 10^{3}$	$E_{2}=-1.0855613023740108\times 10^{5}$	$F_{2}=-1.4333276848124991\times 10^{4}$
$C_{3}=-3.448024120178031\times 10^{2}$	$D_{3}=7.085328250622781\times 10^{4}$	$E_{3}=1.2366226853551846\times 10^{6}$	$F_{3}=1.5844795475466104\times 10^{5}$
$C_{4}=3.3608878187425616\times 10^{3}$	$D_{4}=-6.880294364698327\times 10^{5}$	$E_{4}=-8.525703779146181\times 10^{6}$	$F_{4}=-1.0732374154377037\times 10^{6}$
$C_{5}=-2.16874689789338\times 10^{4}$	$D_{5}=4.38475045543984\times 10^{6}$	$E_{5}=3.811156503092895\times 10^{7}$	$F_{5}=4.751505653867043\times 10^{6}$
$C_{6}=9.585089984945061\times 10^{4}$	$D_{6}=-1.913684709940173\times 10^{7}$	$E_{6}=-1.1474487180812941\times 10^{8}$	$F_{6}=-1.423833477410893\times 10^{7}$
$C_{7}=-2.9756594495868863\times 10^{5}$	$D_{7}=5.878910550570333\times 10^{7}$	$E_{7}=2.3689719428208095\times 10^{8}$	$F_{7}=2.934219487234978\times 10^{7}$
$C_{8}=6.586747075287596\times 10^{5}$	$D_{8}=-1.2907434739579438\times 10^{8}$	$E_{8}=-3.357446782233443\times 10^{8}$	$F_{8}=-4.157826113934839\times 10^{7}$
$C_{9}=-1.0439330743873294\times 10^{6}$	$D_{9}=2.0333119451296386\times 10^{8}$	$E_{9}=3.208894976684707\times 10^{8}$	$F_{9}=3.976891001079562\times 10^{7}$
$C_{10}=1.175145681699476\times 10^{6}$	$D_{10}=-2.2789818435778013\times 10^{8}$	$E_{10}=-1.9753597393059543\times 10^{8}$	$F_{10}=-2.4512920979958445\times 10^{7}$
$C_{11}=-9.169969999477104\times 10^{5}$	$D_{11}=1.7731537748135614\times 10^{8}$	$E_{11}=7.069183538456792\times 10^{7}$	$F_{11}=8.786343590947581\times 10^{6}$
$C_{12}=4.7144049824688485\times 10^{5}$	$D_{12}=-9.099596035342257\times 10^{7}$	$E_{12}=-1.117187473037543\times 10^{7}$	$F_{12}=-1.3909986799395573\times 10^{6}$
$C_{13}=-1.4358936235081303\times 10^{5}$	$D_{13}=2.7689684956188273\times 10^{7}$	-	-
$C_{14}=1.9623293103232572\times 10^{4}$	$D_{14}=-3.7832636200107816\times 10^{6}$	-	-
